# Exploring educational hypogamy among women in urban and rural China: Insights from random forest machine learning

**DOI:** 10.1371/journal.pone.0331744

**Published:** 2025-09-08

**Authors:** Qing Chang, Penghui Wu, Sensen Wang, Ming Zhang

**Affiliations:** 1 Faculty of Educational Sciences, Xinjiang Hetian College, Hetian, China; 2 Center for Studies of Education and Psychology of Ethnic Minorities in Southwest China, Southwest University, Chongqing, China; 3 College of Educational Science, Xinjiang Normal University, Urumqi, China; 4 School of Mathematics and Physics, Xinjiang Hetian College, Hetian, China; University of Pretoria, SOUTH AFRICA

## Abstract

**Background:**

Educational hypogamy, where women marry men with lower educational attainment, reflects evolving gender roles and societal norms. In China, the rapid expansion of education, coupled with persistent traditional values, provides a unique context to study this phenomenon.

**Methods:**

Using data from the 2013, 2015, 2017, 2018, and 2021 waves of the China General Social Survey (CGSS), this study applies logistic regression models and Random Forest machine learning techniques to analyze the impact of education on women’s selection of hypogamy. Key control variables include age, income, parental education, and household registration, with a focus on urban-rural differences.

**Results:**

Between 2013 and 2021, the proportion of women choosing hypogamy increased from 17.42% to 20.06%. As education levels rose, so did the likelihood of choosing hypogamy, particularly among women with higher educational attainment. Other factors such as income and parental education displayed complex interactions with hypogamy, with urban women experiencing more nuanced influences compared to rural women, who showed a clearer education-driven pattern. The random forest analysis further confirmed education as the most significant predictor of hypogamy.

**Discussion/conclusion:**

The rise in educational hypogamy highlights women’s increasing autonomy and challenges to traditional gender norms, especially in rural areas where education’s impact is more pronounced. Urban-rural disparities suggest the need for targeted policies to promote gender equality. Future research should examine the long-term implications of educational hypogamy on household and child-rearing dynamics.

## Introduction

Educational hypogamy, where women marry men with lower educational attainment, has gained increasing scholarly attention as global educational landscapes shift [1,2]. This phenomenon reflects broader societal transformations, including the expansion of women’s educational rights and progress toward gender equality [3,4]. In many countries, educational hypogamy has emerged alongside a narrowing or reversing gender gap in education and a relaxation of traditional gender norms and marriage structures. The decline in educational hypergamy (women marrying men with higher education) and the rise of hypogamy are often seen as markers of a move toward more egalitarian family dynamics, challenging entrenched norms of male dominance in marriage [5]. These changes underscore the importance of examining how societal values, gender roles, and education intersect in shaping marriage patterns.

In China, the interplay between traditional gender norms and rapid educational expansion provides a unique context for studying educational hypogamy [6]. Historically, the ideal of “male superiority and female inferiority” has dominated social and cultural narratives, reflecting deep-rooted patriarchal values [7]. Moreover, men in China have traditionally enjoyed higher average years of education compared to women [8]. However, recent decades have witnessed remarkable progress in women’s educational attainment. For instance, female participation in higher education has not only increased but now exceeds that of men, marking a significant reversal in the educational gender gap [9]. Despite these advancements, societal adherence to traditional gender norms remains strong, raising questions about the implications of educational hypogamy in the Chinese context. How should we interpret the rise of this marital pattern amidst persistent gender norms that emphasize male dominance?

This study addresses these questions by analyzing trends and factors influencing educational hypogamy in urban and rural China using data from the China General Social Survey (CGSS) for 2013, 2015, 2017, 2018, and 2021. First, we investigate the trends and variations in educational hypogamy across different educational strata. Second, we employ a logistic regression model to explore potential drivers of educational hypogamy, such as individual, societal, and structural factors. Finally, we examine regional differences between urban and rural areas to assess how local contexts shape patterns of educational hypogamy. By integrating these dimensions, our research provides a comprehensive understanding of educational hypogamy in China and its broader social implications.

## Literature review

### Research on educational hypogamy

Educational hypogamy, where women marry partners with lower educational attainment, has garnered increasing scholarly attention in recent decades as gender dynamics and societal structures evolve globally. The global trend of educational hypogamy emerges as a byproduct of women’s educational advancements and the decline of traditional hypergamous patterns occurring concurrently [10,11]. The expansion of female education, particularly in contexts where women’s attainment surpasses that of men, has significantly altered marriage market dynamics [12]. In Western contexts, shifts in educational distribution and changes in societal norms have been major drivers of this trend [13,14]. However, the extent to which this transition reflects deeper transformations in gender norms versus demographic changes remains debated [15].

Traditional theories, such as the status exchange framework [16,17], propose that women may compensate for their partner’s lower education with other desirable traits like economic resources or social prestige [18]. These frameworks, however, often emphasize Western contexts where individualism and gender egalitarianism are more pronounced, raising questions about their applicability to non-Western societies, such as China, where cultural norms and structural conditions differ significantly.

### The influence of education on women’s educational hypogamy

The relationship between women’s educational attainment and marital patterns is multifaceted. Higher educational attainment enhances women’s socioeconomic standing and agency, granting them greater bargaining power in the marriage market [19]. This increased agency often diminishes reliance on hypergamous marriages, allowing women to prioritize non-economic criteria, such as shared values or compatibility [20,21]. The shift from a breadwinner-homemaker ideal to more egalitarian partnerships, particularly in Western societies, has further facilitated this transition [22,23].

Nonetheless, the economic empowerment linked to rising female education is not universally consistent. In developing countries like India, limited employment opportunities for educated women have constrained their ability to translate educational gains into economic independence, perpetuating traditional assortative mating patterns [24]. Furthermore, women’s pursuit of degrees in lower-paying fields, such as the humanities, complicates the link between education and marital choices [25].

When women attain higher education in contexts where men’s educational attainment stagnates or increases at a slower pace, the pool of “educationally superior” men narrows. This structural mismatch in the marriage market—known as the marriage market squeeze—can lead highly educated women to reconsider rigid educational criteria and prioritize compensatory attributes in potential partners, such as income, social status, or occupational prestige [10,26]. In this situation, women may choose to “marry down” educationally while still maintaining overall parity or advantage in other domains, leading to an increase in educational hypogamy, especially in high-education societies like the U.S., Nordic countries, or parts of East Asia undergoing gender-role liberalization [27–29].

In contrast, in countries with relatively lower overall educational attainment and persistent gender hierarchies, rising female education may actually intensify women’s pursuit of hypergamous matches. This is because education remains a key marker of male desirability in such settings, and social norms may discourage women from “marrying down” [30,31]. In patriarchal societies, even highly educated women may continue to seek men with equal or higher education levels, perceiving educational homogamy or hypergamy as essential to maintaining social respectability or marital stability [31].

Gender norms play a crucial role in shaping these patterns. In more egalitarian societies, women may have greater flexibility to prioritize traits other than education in partner selection and may not face significant stigma in educationally hypogamous unions. However, in strongly patriarchal contexts, marrying a less-educated man may be perceived as threatening to normative gender roles and thus avoided, regardless of the woman’s own qualifications [32,33].

The effect of education on women’s likelihood of entering educationally hypogamous marriages is not unidirectional. It is mediated by labor market structures, prevailing gender ideologies, cultural norms around marriage, and broader social capital considerations. As such, while education can empower women and increase the likelihood of hypogamy under certain circumstances, it can also reinforce traditional assortative mating preferences in others.

### Cultural and structural contexts in China

In China, educational attainment plays a pivotal role in shaping both individual preferences and structural opportunities in mate selection [34,35]. Since the economic reforms of 1978, education has become a critical marker of social status and human capital [36]. Gender gaps in education have narrowed, and prolonged schooling has contributed to increased educational homogamy in first marriages [31,37]. However, the context-specific interplay between education, cultural norms, and economic opportunities reveals complex dynamics in educational hypogamy.

Urban and rural distinctions significantly influence the marital implications of women’s education. In urban areas, economic modernization and the decline of patriarchal norms have enabled some women to leverage their education for greater economic independence and more egalitarian marital [35]. In contrast, entrenched gender hierarchies and limited employment opportunities in rural areas perpetuate traditional hypergamous patterns [38]. Confucian traditions, emphasizing male dominance and patrilineality, continue to influence marriage practices and gender roles, particularly in rural settings [39–41].

Moreover, women who marry less-educated men often encounter social stigma, as such unions challenge deeply ingrained gender hierarchies. This is especially pronounced in urban areas, where women’s higher education correlates with delayed marriage or remaining single [42]. In rural contexts, societal pressures to conform to traditional norms further discourage hypogamous marriages, even among educated women [43].

### Marriage as a changing institution

Globally, marriage has transitioned from a model of gender specialization to more egalitarian partnerships [44,45]. Historically, theories like Becker’s (1974) exchange theory posited that marriages maximized gains when men’s market advantages complemented women’s domestic roles [46]. However, with the rise of egalitarian values, these traditional dynamics have shifted. Today, young people increasingly prioritize status equality in mate selection [47,48].

In China, this institutional shift is tempered by unique cultural and structural factors. Educational institutions—as spaces for dating and mate selection—reinforce educational homogamy [49,50]. However, disparities in economic benefits between urban and rural areas highlight the uneven transformative potential of education. While urban women may achieve economic independence, rural women’s limited access to professional networks and high-paying jobs constrains their marital choices, perpetuating traditional patterns [38].

The literature underscores that rising female education, while globally significant, interacts with cultural and structural factors to shape marital patterns. In China, the distinct interplay between education, gender norms, and economic opportunities creates a unique context for studying educational hypogamy. Addressing structural barriers and entrenched cultural norms is crucial for fostering gender equality and enabling women to fully realize the transformative potential of education in both urban and rural settings.

### Data, variables and methods

#### Data.

Multi-order stratified probabilistic sampling was applied to data extracted from the 2013, 2015, 2017, 2018, and 2021 waves of the China General Social Surveys (CGSS2013, CGSS2015, CGSS2017, CGSS2018, and CGSS2021). These datasets represent some of the most comprehensive and representative sources of information on the Chinese population, including variables relevant to the topic of education and hypogamy. Since its inception in 2003, the CGSS has periodically collected data, with intervals ranging from one to several years. For this study, data from these five survey years were selected, with a focus on CGSS2013 and CGSS2021, spanning a time frame of approximately eight years.

### Variables

#### Dependent variable.

The dependent variable in this study is hypogamy, which refers to a marriage where the wife’s educational attainment exceeds that of her husband. This variable is derived from responses to the following two survey questions: Q1:”What is your highest level of educational attainment?” and Q2:”What is your spouse or cohabiting partner’s highest level of educational attainment?”

To facilitate consistent measurement, we converted reported educational levels into standardized years of education based on established guidelines. For example, completing high school equates to 12 years, a bachelor’s degree to 16 years, and a master’s degree to 19 years. Using this numerical scale, a marriage is classified as hypogamous and coded as 1 when the wife’s total years of education exceed the husband’s. Conversely, if the wife’s years of education are equal to or lower than the husband’s, the variable is coded as 0 [51].

#### Independent variable.

The independent variable in this study is education. Education can generally be measured in two ways: as a numerical variable representing “years of education” or as a categorical variable reflecting “education level.” Researchers commonly categorize education levels into seven groups: primary school and below, middle school, high school, vocational high school/technical school, technical secondary school, junior college, and bachelor’s degree or above.

In the Chinese context, while high school education is typically regarded as lower than vocational high school or technical school, which are in turn considered lower than technical secondary school, these three types of education share similarities. All primarily admit students from junior high schools and follow a three-year curriculum, making them equivalent in terms of educational level. Consequently, this study grouped “high school,” “vocational high school/technical school,” and “technical secondary school” into a single category. Education levels were thus classified into five categories: primary school and below, middle school, high school (including high school, vocational high school, technical school, and technical secondary school), junior college, and bachelor’s degree or above [52].

#### Control variables.

In addition to education, previous studies have shown that various factors significantly influence individual outcomes, including age, income, parental education levels, family social status at age 14 and household registration (hukou) [53–56]. Research has also indicated that the relationship between age and certain outcomes, such as well-being, is often U-shaped rather than linear (Hayo, 2007). To account for this, the square of age is frequently included in models. Similarly, due to the skewed distribution of income, the natural logarithm of income is often used in analyses.

Based on these considerations, the control variables in this study include the following: age, age squared, income (log-transformed), parental education levels (classified into five categories: primary school and below, middle school, high school [including high school, vocational high school, technical school, and technical secondary school], junior college, and bachelor’s degree or above), family social status at age 14 (measured on a 10-point scale ranging from 1 for the lowest to 10 for the highest), and household registration (1 = “urban,” 0 = “rural”).

## Methods

This study sought to determine the relationships between education and hypogamy in the decade from 2013 to 2021 in a Chinese population. To examine the determinants of hypogamy, we adopted a two-pronged methodological approach combining Logistic Regression and Random Forest machine learning.

1. Logistic regression model

Given that the key dependent variable—hypogamy—is binary (1 = hypogamy, 0 = non-hypogamy), a Logistic Regression model was employed to estimate the effect of education and other individual characteristics on the likelihood of selecting a hypogamous marriage. The simplified regression model can be expressed as::


$$Logit(Hypogamy_{i})=\beta_{0}+\beta_{1}Education_{i}+\beta_{2}X_{i}+\varepsilon_{i}$$ (1)

Where:$Hypogamy_{i}$: Binary dependent variable (1 = hypogamy, 0 = non-hypogamy); $Education_{i}$: Independent variable representing the respondent’s education level; $X_{i}$: Vector of control variables; $\beta_{0}$: Intercept; $\beta_{1}\;and\;\beta_{2}$: Coefficients of the independent variable and control variables; $\varepsilon_{i}$: Error term.

2. Random forest regression and robustness analysis

To complement the Logit analysis and assess the relative importance and non-linear relationships between predictors and hypogamy, we applied Random Forest regression, a non-parametric machine learning method known for its robustness in handling high-dimensional data and complex interactions.

Random Forest is particularly well-suited for capturing variable interactions and addressing multicollinearity issues that may confound traditional regression models. We employed two standard metrics to evaluate variable importance:

Mean Decrease Accuracy (MDA): Measures the decrease in model accuracy when a given variable is removed, reflecting its predictive value.

Mean Decrease Gini (MDG): Reflects the contribution of each variable to the reduction of node impurity (Gini index) in the decision trees.

Additionally, we generated Partial Dependence Plots (PDPs) to visualize the marginal effects of education levels on the probability of hypogamy while averaging out the effects of all other variables. This allowed us to observe how the impact of education varies across its levels and between years (2013 and 2021), revealing changes in the education–marriage relationship over time.

## Findings

### Changes in women’s selection of hypogamy across different education levels from 2013 to 2021

First, a preliminary analysis was conducted to explore the changes in women’s selection of hypogamy in different education levels over the period from 2013 to 2021. Here, we test the differences in the proportion of hypogamy choices for different education levels, as shown in Table 1.

**Table 1:** The proportions of choosing hypogamy in different educational attainment levels across different years.

	2013	2015	2017	2018	2021
primary school and below	3.28%	4.73%	3.50%	3.77%	5.89%
middle school	15.71%	17.39%	17.20%	15.93%	18.18%
high school	38.15%	34.36%	38.08%	36.18%	42.86%
junior college	40.34%	38.06%	31.61%	32.96%	38.21%
bachelor’s degree or above	39.51%	37.72%	36.48%	31.42%	37.33%
total	17.42%	16.93%	17.76%	16.67%	20.06%

It was found that compared to 2013, there had been certain changes in the overall proportion of women choosing hypogamy by 2021. The total proportion of women selecting hypogamy increased from 17.42% in 2013 to 20.06% in 2021. When divided into different education levels, different trends could be observed. For the group with primary school education and below, the proportion showed an upward trend over the years, rising from 3.28% in 2013 to 5.89% in 2021. The middle school group also had an increasing trend with the proportion growing from 15.71% in 2013 to 18.18% in 2021. The high school group’s proportion fluctuated but generally had an upward tendency as well, increasing from 38.15% in 2013 to 42.86% in 2021. However, for the junior college group and the group with a bachelor’s degree or above, their proportions fluctuated with some years showing decreases and then rebounds, yet still presented certain variations over the time span.

Notably, as women’s education levels improved, the proportion of them choosing hypogamy also tended to increase. For example, in 2013, the proportion of women with a primary school education and below choosing hypogamy was 3.28%, while for those with a middle school education, it was 15.71%, nearly five times that of the former. Moving up the education ladder, women with a high school education had a proportion of 38.15%, which was more than double that of the middle school group. And among those with a junior college degree or above, the proportion in 2013 was 40.34% and 39.51% respectively, remaining at a relatively high level compared to the lower education groups. This clear upward trend in the proportion with increasing education levels indicates a potential relationship between educational attainment and the choice of hypogamy.

### Overall effect of education on women’s selection of hypogamy in China

Table 2 presents the results of the logistic Regression analysis, which examines the overall effect of education on women’s selection of hypogamy in China. The findings indicate that age has a significant positive effect on hypogamy in 2013, but this effect weakens and becomes statistically insignificant by 2021. Similarly, the square of age showed a significant negative relationship in 2013, suggesting a nonlinear relationship, but this effect diminished over time.

**Table 2:** Logistic regression analysis.

	2013		2021	
	hypogamy	hypogamy	hypogamy	hypogamy
age	0.0753^***^ (0.0225)	0.0528^**^ (0.0225)	−0.0036 (0.0252)	−0.0285 (0.0256)
age^2^	−0.0006^***^ (0.0002)	−0.0003 (0.0002)	0.0002 (0.0002)	0.0005^*^ (0.0002)
income	0.0115 (0.0450)	0.0059 (0.0457)	−0.0706 (0.0557)	−0.1122^*^ (0.0574)
Father’s education	−0.0957^***^ (0.0285)	−0.0945^***^ (0.0277)	−0.0743^**^ (0.0348)	−0.0836^**^ (0.0351)
Mother’s education	−0.0314 (0.0341)	0.0081 (0.0332)	−0.1053^**^ (0.0437)	−0.0674 (0.0438)
Social states at age 14	−0.0516^*^ (0.0269)	−0.0847^***^ (0.0271)	−0.0443 (0.0278)	−0.0725^**^ (0.0283)
household registration	0.5602^***^ (0.1165)	0.7458^***^ (0.1177)	0.4354^***^ (0.1292)	0.5668^***^ (0.1299)
Education levels	1.0693^***^ (0.0538)		0.8812^***^ (0.0581)	
Primary school and below				
Middle school		2.2116^***^ (0.1702)		1.7739^***^ (0.1817)
High school		3.7949^***^ (0.1871)		3.3219^***^ (0.2041)
Junior college		4.2135^***^ (0.2284)		3.4429^***^ (0.2514)
Bachelor and above		4.4041^***^ (0.2499)		3.5763^***^ (0.2669)
Constant	−5.9468^***^ (0.6092)	−5.6004^***^ (0.5928)	−3.5506^***^ (0.7260)	−2.9919^***^ (0.7091)
N	4200	4200	2671	2671
Pseudo R^2^	0.1508	0.1953	0.1132	0.1567

Note: *, **, and ***, respectively denote the 10%, 5%, and 1% significance levels. The values in parentheses are standard errors.

Income showed no significant relationship with hypogamy in 2013 but became significantly negative in 2021, indicating that higher income levels may reduce the likelihood of women selecting hypogamy in more recent years.

Parental education levels had varying effects. In 2013, father’s education was found to have a significant negative relationship with hypogamy, and this persisted into 2021. However, mother’s education, while insignificant in 2013, became a significant factor in reducing hypogamy by 2021.

The family’s social status at age 14 consistently showed a significant negative relationship with hypogamy across both years, suggesting that higher social status during childhood reduces the likelihood of selecting hypogamy.

Household registration (urban vs. rural) demonstrated a robust and significant positive relationship with hypogamy in both 2013 and 2021, with urban women being less likely to select hypogamy.

Education level emerged as the strongest determinant of hypogamy. In both years, higher education levels were associated with a significantly increased likelihood of selecting hypogamy, and the magnitude of this effect grew with higher levels of education. Specifically, women with bachelor’s degrees or above were the most likely to select hypogamy, followed by those with junior college, high school, and middle school education levels.

When the education variable (edu) was modeled as a categorical variable using i.edu in the second and fourth columns, noticeable changes were observed in the significance levels and coefficients of other variables. For instance, variables such as income, family’s social status, and parental education exhibited shifts in their statistical significance and effect sizes. This suggests that these variables are interlinked with education’s impact on hypogamy. By categorizing education, the model better captures the nuanced and interactive effects between education and other socioeconomic factors.

This finding highlights that the influence of variables like income and social background on hypogamy cannot be fully understood without considering the role of education. Education, particularly when categorized into detailed levels, appears to mediate or interact with these factors, reinforcing its central role in shaping marital preferences. This underscores the complexity of marital decision-making and the interplay of education with broader socioeconomic determinants in influencing women’s selection of hypogamy in China.

### Analysis of the importance of factors influencing the selection of hypogamy

To ensure reproducibility and model reliability, this study constructed Random Forest models using data from both 2013 (N = 4,200) and 2021 (N = 2,671). A fixed random seed of 123 was applied across all procedures. For each dataset, a consistent sampling strategy was employed: 75% of the data was randomly assigned to the training set and 25% to the test set, resulting in 3,150 training and 1,050 test cases for 2013, and 2,003 training and 668 test cases for 2021. The test set was reserved exclusively for final evaluation, ensuring that no information from it influenced model training or hyperparameter selection..

Given the significant class imbalance in the target variable hypogamy, the training data underwent up-sampling using the upSample method to balance the two outcome categories at a 1:1 ratio, thereby mitigating potential model bias toward the majority class. Hyperparameter tuning was conducted via five-fold cross-validation within the training set. Specifically, the number of variables randomly sampled at each split (mtry) was optimized through a grid search over the range from 1 to the square root of the total number of features. Other hyperparameters were set as follows: number of trees at 500, minimum samples per leaf node at 1, minimum samples required to split a node at 2, and unrestricted maximum tree depth. The optimal mtry value for both datasets was found to be 3, at which point the model’s error rate stabilized. With these parameters, the models demonstrated satisfactory performance: for the 2013 dataset, the out-of-bag (OOB) error rate was 5.99%, and the test set accuracy reached 78.57% (error rate 21.43%). For the 2021 dataset, the OOB error rate was 8.21%, and the test set accuracy was 74.85% (error rate 25.15%). These results indicate that both models achieved stable and acceptable predictive performance, supporting their use in subsequent variable importance analysis.

In this study, we utilized Random Forest regression to assess the relative importance of various factors influencing women’s decisions regarding hypogamy (marrying down in terms of education). The Random Forest algorithm is particularly valuable for understanding the complex, non-linear relationships between variables, as it accounts for interactions among predictors that may not be evident in traditional regression models. Two distinct measures of variable importance were employed: Mean Decrease Accuracy and Mean Decrease Gini. The former evaluates how much the accuracy of the model decreases when a variable is removed, while the latter assesses the contribution of each variable to the Gini impurity reduction in the decision trees, reflecting how well each variable helps to classify the outcome.

**Fig 1:**
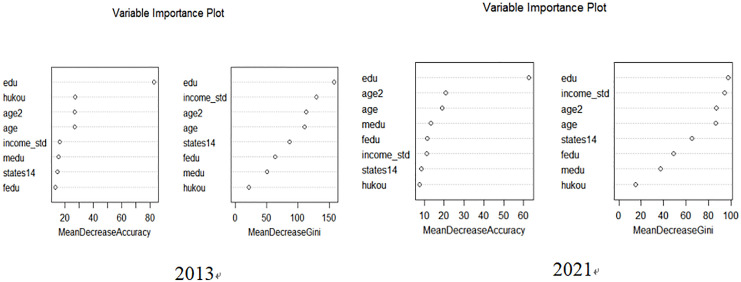
Results of random forest regression.

The results in Fig 1 from both 2013 and 2021 indicate that education (edu) consistently ranks as the most influential factor in determining hypogamy. In 2013, both Mean Decrease Accuracy and Mean Decrease Gini highlighted education as the primary determinant, followed by household registration status (hukou), and age-related variables. These findings suggest that education is central to the decision-making process for women regarding marital choices, and factors such as household registration and age also play significant roles. In 2021, the importance ranking remained largely unchanged, with education continuing to dominate, followed by income (income_std), and age-related factors. This consistency across the years underscores the lasting impact of education on women’s reproductive and marital preferences.

The importance of socioeconomic variables such as income and parental education also emerged from the analysis, though they were secondary to education. These findings are consistent with the primary regression results, where education was identified as the most significant determinant of hypogamy. The Random Forest analysis further strengthens the robustness of these conclusions, illustrating that education, along with socio-economic status and demographic factors, continues to be crucial in shaping women’s marital decisions in both urban and rural contexts.

**Fig 2:**
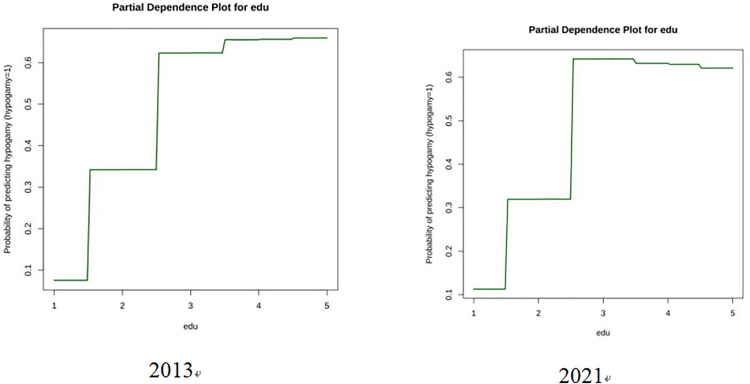
Partial dependence plots.

In this study, partial dependence plots in Fig 2 were employed to analyze the relationship between hypergamy and education level (edu) using the random forest machine – learning method. The horizontal axis of the plots represents different education levels, where 1 indicates “Primary school and below”, 2 is “Middle school”, 3 is “High school”, 4 is “Junior college”, and 5 is “Bachelor and above”. Unlike traditional linear regression, which only shows the influence of independent variables on dependent variables in terms of average trends through regression coefficients, random forest regression with partial dependence plots can vividly illustrate how changes in the independent variable at various levels affect the trend of the dependent variable.

The partial dependence plots for 2013 and 2021 reveal interesting insights. In 2013, as the education level increased, the tendency of hypergamy showed a significant downward trend, especially when moving from lower to intermediate education levels. However, when the education level reached high school and above, the decrease in the hypergamy tendency slowed down. By 2021, although the overall pattern remained a negative correlation between education level and hypergamy, the hypergamy tendency at each education level was generally lower compared to 2013. The decline was more gradual in the early stages of education level improvement, suggesting that over time, the relationship between education and hypergamy selection has evolved. This indicates that education level is an important factor influencing hypergamy, and its impact may change over different time periods.

### Robustness test

To ensure the robustness of our findings, we conducted several sensitivity analyses in Tables 3 and 4. First, we focused exclusively on the Han ethnic group, which constitutes the majority population and is more influenced by Confucian cultural traditions. This subgroup analysis aimed to determine whether our primary results held consistent within this culturally homogeneous group.

**Table 3:** Robustness analysis of adjusting the sample size and changing the way of assigning values to the dependent variable.

	Select the Han ethnic group as the sample.				Change the way of assigning values to the dependent variable.			
	2013		2021		2013		2021	
age	0.0853^***^ (0.0238)	0.0599^**^ (0.0237)	−0.0078 (0.0262)	−0.0293 (0.0266)	0.0156 (0.0134)	0.0174 (0.0134)	−0.0040 (0.0184)	−0.0054 (0.0184)
age^2^	−0.0007^***^ (0.0002)	−0.0004 (0.0002)	0.0002 (0.0002)	0.0005^*^ (0.0003)	−0.0001 (0.0001)	−0.0002 (0.0001)	0.0001 (0.0002)	0.0002 (0.0002)
income	0.0126 (0.0460)	0.0079 (0.0437)	0.0003 (0.0004)	−0.1252^*^ (0.0474)	0.0145 (0.0460)	0.0159 (0.0257)	0.0003 (0.0002)	−0.1145^*^ (0.0546)
Father’s education	−0.0882^***^ (0.0292)	−0.0891^***^ (0.0285)	−0.0670^*^ (0.0359)	−0.0771^**^ (0.0363)	−0.0873^***^ (0.0190)	−0.0863^***^ (0.0190)	−0.0906^***^ (0.0246)	−0.0927^***^ (0.0246)
Mother’s education	−0.0224 (0.0355)	0.0148 (0.0345)	−0.1057^**^ (0.0459)	−0.0658 (0.0459)	−0.0553^**^ (0.0240)	−0.0555^**^ (0.0241)	−0.0615^*^ (0.0314)	−0.0574^*^ (0.0315)
Social states at age 14	−0.0641^**^ (0.0281)	−0.0948^***^ (0.0283)	−0.0522^*^ (0.0291)	−0.0788^***^ (0.0295)	−0.0422^**^ (0.0172)	−0.0427^**^ (0.0173)	−0.0461^**^ (0.0195)	−0.0495^**^ (0.0196)
household registration	0.5178^***^ (0.1202)	0.7227^***^ (0.1223)	0.4291^***^ (0.1336)	0.5580^***^ (0.1346)	0.6422^***^ (0.0719)	0.6479^***^ (0.0723)	0.6908^***^ (0.0909)	0.6999^***^ (0.0911)
Education levels	1.0496^***^ (0.0556)		0.8940^***^ (0.0608)		0.8802^***^ (0.0349)		0.8482^***^ (0.0416)	
Primary and below								
Middle school		2.2010^***^ (0.1810)		1.6931^***^ (0.1872)		0.9374^***^ (0.0768)		1.0012^***^ (0.0979)
High school		3.8120^***^ (0.1973)		3.2472^***^ (0.2102)		1.6852^***^ (0.0983)		1.7998^***^ (0.1267)
Junior college		4.2081^***^ (0.2392)		3.4163^***^ (0.2603)		3.0091^***^ (0.1412)		2.7941^***^ (0.1741)
Bachelor and above		4.3617^***^ (0.2613)		3.5886^***^ (0.2768)		3.3399^***^ (0.1590)		3.2645^***^ (0.1837)
Constant	−6.1576^***^ (0.6462)	−5.7946^***^ (0.6267)	−3.4944^***^ (0.7590)	−2.9354^***^ (0.7402)	−2.6691^***^ (0.3664)	−1.8659^***^ (0.3595)	−2.3252^***^ (0.5205)	−1.5401^***^ (0.5085)
N	3845	3845	2474	2474	4200	4200	2671	2671
R^2^/pseudo R^2^	0.1482	0.1948	0.1143	0.1530	0.1533	0.1564	0.1473	0.1495

Note: *, **, and ***, respectively denote the 10%, 5%, and 1% significance levels. The values in parentheses are standard errors.

**Table 4:** Propensity Score Matching (PSM) to address self -selection bias.

2013							
Divide into two sample sizes according to whether one has received higher education.							
Variable	Sample	Treated	Controls	Difference	S.E.	T-stat	
hypogamy	Unmatched	0.397313	0.143789	0.253524	0.017365	14.6	Radius Matching, K = 0.5
	ATT	0.401163	0.176357	0.224806	0.035929	6.26	
hypogamy	Unmatched	0.397313	0.143789	0.253524	0.017365	14.6	Nearest Neighbor Matching
	ATT	0.397313	0.174664	0.222649	0.036525	6.1	
hypogamy	Unmatched	0.397313	0.143789	0.253524	0.017365	14.6	Kernel Matching
	ATT	0.399614	0.187548	0.212066	0.027739	7.65	
Divide the data into two sample sizes: a high – education group and a low – education group, with the boundary set as the mean value of education in the total sample.							
Variable	Sample	Treated	Controls	Difference	S.E.	T-stat	
hypogamy	Unmatched	0.386994	0.084382	0.302612	0.011919	25.39	Radius Matching, K = 0.5
	ATT	0.386994	0.092784	0.294211	0.023577	12.48	
hypogamy	Unmatched	0.386994	0.084382	0.302612	0.011919	25.39	Nearest Neighbor Matching
	ATT	0.386994	0.092784	0.294211	0.023577	12.48	
hypogamy	Unmatched	0.386994	0.084382	0.302612	0.011919	25.39	Kernel Matching
	ATT	0.386994	0.086392	0.300602	0.020791	14.46	
2021							
Divide into two sample sizes according to whether one has received higher education.							
Variable	Sample	Treated	Controls	Difference	S.E.	T-stat	
hypogamy	Unmatched	0.368932	0.170872	0.19806	0.021149	9.37	Radius Matching, K = 0.5
	ATT	0.372549	0.20098	0.171569	0.042271	4.06	
hypogamy	Unmatched	0.368932	0.170872	0.19806	0.021149	9.37	Nearest Neighbor Matching
	ATT	0.368932	0.199029	0.169903	0.04313	3.94	
hypogamy	Unmatched	0.368932	0.170872	0.19806	0.021149	9.37	Kernel Matching
	ATT	0.368932	0.18138	0.187552	0.034805	5.39	
Divide the data into two sample sizes: a high – education group and a low – education group, with the boundary set as the mean value of education in the total sample.							
Variable	Sample	Treated	Controls	Difference	S.E.	T-stat	
hypogamy	Unmatched	0.393904	0.111111	0.282793	0.015726	17.98	Radius Matching, K = 0.5
	ATT	0.393904	0.071512	0.322392	0.026503	12.16	
hypogamy	Unmatched	0.393904	0.111111	0.282793	0.015726	17.98	Nearest Neighbor Matching
	ATT	0.393904	0.071512	0.322392	0.026503	12.16	
hypogamy	Unmatched	0.393904	0.111111	0.282793	0.015726	17.98	Kernel Matching
	ATT	0.393904	0.07321	0.320694	0.025092	12.78	

Second, we redefined the dependent variable, hypogamy, by calculating the difference between the educational attainment years of females and their male partners. This alternative measurement allowed us to assess the impact of educational disparity on hypogamy more directly.

Third, we employed Propensity Score Matching (PSM) to address potential self-selection bias in women’s educational attainment. By matching individuals with similar characteristics—such as age, income, parental education levels, family social status at age 14, and household registration status—we aimed to isolate the effect of education on hypogamy. Specifically, we divided the sample into high and low education groups based on the mean educational level and applied PSM within these strata.

The results from these robustness checks were consistent with our main regression findings, reinforcing the reliability of our conclusions.

### Urban-Rural differences in the education effect on women’s selection of hypogamy in China

As shown in Table 5, the regression analysis for rural and urban household registration samples reveals urban-rural differences in the education effect on women’s selection of hypogamy. The results indicate that education level had a significant positive effect on hypogamy for both rural and urban women; however, the positive effect was greater in rural areas than in urban areas.

**Table 5:** Analysis of urban – rural heterogeneity.

	rural		urban		rural		urban	
	2013				2021			
age	0.0795^**^ (0.0322)	0.0484 (0.0317)	0.0942^***^ (0.0326)	0.0517 (0.0331)	0.0601 (0.0371)	0.0199 (0.0370)	−0.0235 (0.0374)	−0.0870^**^ (0.0395)
age^2^	−0.0005 (0.0003)	−0.0001 (0.0003)	−0.0008^***^ (0.0003)	−0.0004 (0.0003)	−0.0004 (0.0004)	0.0000 (0.0004)	0.0004 (0.0003)	0.0010^***^ (0.0004)
income	−0.0095 (0.0658)	−0.0245 (0.0673)	0.0454 (0.0636)	0.0290 (0.0634)	−0.1126 (0.0735)	−0.1218^*^ (0.0729)	−0.0768 (0.1066)	−0.1084 (0.1096)
Father’s education	−0.1006^*^ (0.0516)	−0.1118^**^ (0.0509)	−0.0878^***^ (0.0336)	−0.0870^***^ (0.0333)	0.0116 (0.0527)	−0.0129 (0.0532)	−0.1201^***^ (0.0465)	−0.1271^***^ (0.0474)
mother’s education	0.0410 (0.0685)	0.0428 (0.0668)	−0.0160 (0.0389)	−0.0052 (0.0386)	−0.1417^*^ (0.0726)	−0.1152 (0.0714)	−0.0575 (0.0554)	−0.0381 (0.0565)
Social states at age 14	−0.0495 (0.0425)	−0.0676 (0.0428)	−0.0667^*^ (0.0347)	−0.0873^**^ (0.0351)	−0.0845^**^ (0.0393)	−0.0936^**^ (0.0394)	−0.0155 (0.0399)	−0.0480 (0.0411)
Education levels	1.6983^***^ (0.1005)		0.7749^***^ (0.0630)		1.1733^***^ (0.0892)		0.6557^***^ (0.0766)	
Primary school and below								
Middle school		2.4475^***^ (0.2103)		1.6786^***^ (0.3622)		1.7787^***^ (0.2077)		1.7037^***^ (0.4643)
High school		4.2587^***^ (0.2499)		3.0716^***^ (0.3519)		3.3316^***^ (0.2549)		3.2994^***^ (0.4616)
Junior college		4.6750^***^ (0.4048)		3.5045^***^ (0.3728)		3.5349^***^ (0.3463)		3.3731^***^ (0.4944)
Bachelor and above		4.2865^***^ (0.6112)		3.7095^***^ (0.3862)		4.0035^***^ (0.4219)		3.3773^***^ (0.4947)
Constant	−7.1378^***^ (0.8414)	−5.3701^***^ (0.7854)	−5.3726^***^ (0.8632)	−4.6896^***^ (0.8597)	−5.3376^***^ (1.0236)	−3.7076^***^ (0.9561)	−2.4685^**^ (1.0488)	−1.4046 (1.0824)
N	2457	2457	1743	1743	1679	1679	992	992
Pseudo R^2^	0.2114	0.2383	0.1020	0.1271	0.1500	0.1726	0.0794	0.1245

Note: *, **, and ***, respectively denote the 10%, 5%, and 1% significance levels. The values in parentheses are standard errors.

In rural areas, every increase in education level was associated with a stronger likelihood of selecting hypogamy. Women with middle school, high school, junior college, and bachelor’s degrees or above were all significantly more likely to select hypogamy compared to those with only primary school or below education. The magnitude of this effect increased progressively with higher levels of education, showing a clear and robust pattern.

In urban areas, education also had a significant positive relationship with hypogamy, but the effect was comparatively smaller than that in rural areas. Urban women with higher education levels were more likely to select hypogamy, but the increase in likelihood across education levels was less pronounced than in rural areas. For instance, the coefficients for bachelor’s degrees and above were slightly lower in urban areas compared to rural areas, suggesting that the influence of higher education on hypogamy is moderated by urban-specific factors.

When subdivided into specific education levels, more detailed findings emerged. For rural women, the likelihood of selecting hypogamy increased substantially as education level rose, with those holding junior college or bachelor’s degrees exhibiting the strongest effects. In contrast, for urban women, while the pattern was consistent, the differences between middle school and higher education levels were relatively smaller, reflecting a more homogeneous influence of education on hypogamy in urban contexts.

Other variables also showed notable differences between rural and urban samples. Father’s education had a consistently negative relationship with hypogamy in both contexts, but the effect was slightly stronger in rural areas. Additionally, family social status at age 14 was a significant negative predictor of hypogamy for urban women, while its influence was weaker or insignificant in rural areas.

The use of i.edu to categorize education levels provided further insights. After categorization, changes in the significance levels and coefficients of variables like income, father’s education, and family social status indicated that these factors interact with education to influence hypogamy. This suggests that the education effect on hypogamy is not isolated but intertwined with other socioeconomic characteristics, particularly in urban areas where these interactions appear more complex.

Overall, the results demonstrate that while education positively affects women’s selection of hypogamy across both rural and urban contexts, its influence is stronger and more pronounced in rural areas. The findings highlight the critical role of education in shaping marital preferences and underscore the need to account for urban-rural disparities when analyzing the effects of socioeconomic factors on hypogamy.

## Conclusions and discussion

This study explored the dynamics of educational hypogamy in China, analyzing data from the China General Social Survey (CGSS) spanning 2013–2021. Our findings reveal several key insights into how education and other socioeconomic factors are associated with women’s marital preferences across urban and rural contexts.

First, the study found a steady increase in the overall proportion of women selecting educational hypogamy over the observed period, rising from 17.42% in 2013 to 20.06% in 2021. This trend underscores the ongoing transformation of marital patterns in China, reflecting the dual forces of expanding educational opportunities for women and evolving societal norms. Notably, women with higher levels of education tended to be more likely to form hypogamous unions than those with lower levels of education, suggesting a shift in how educational attainment correlates with marital choices. This pattern may indicate that women with bachelor’s degrees or above are more open to considering partners with lower education, possibly due to a reevaluation of traditional matching criteria.

Second, the Logistic Regression analysis revealed that education was positively associated with hypogamy, and this association appeared to be stronger at higher levels of education. However, other factors also demonstrated complex interactions with educational attainment. For example, higher income levels were negatively associated with hypogamy in 2021, which may imply that greater financial independence is linked to a reduced likelihood of marrying a less-educated partner. Similarly, parental education and childhood social status negatively influenced hypogamy, suggesting that family background continues to shape marital preferences. These findings align with the broader literature on the interplay of socioeconomic variables in marital decision-making.

Third, the urban – rural divide in the relationship between education and hypogamy was notably pronounced. Rural women exhibited a much stronger positive correlation between education and hypogamy compared to their urban counterparts, a phenomenon that can be elucidated by multiple factors related to gender-specific educational landscapes and marriage markets. In rural areas, there has long been a significant gender gap in educational attainment. Historically, boys in rural regions have often received more educational support and opportunities compared to girls. As a result, when rural women manage to obtain higher education, they are in a relatively small-sized group within their local marriage market. This scarcity of highly – educated rural women means that they face a limited pool of potential partners with similar educational backgrounds. Moreover, the rural marriage market has unique characteristics. Marriage in rural areas is often closely tied to local social networks and family – based arrangements. The limited mobility of rural residents also restricts the scope of potential partners. For educated rural women, finding a local partner with an equivalent educational level becomes even more challenging. In this situation, hypogamy may seem like a more feasible option. They may choose partners with lower education levels, perhaps from the same local area, to maintain family – based social connections and the stability of rural life. In contrast, urban areas present a different scenario. The educational gender gap in urban regions is much narrower, and both men and women have relatively equal access to a wide range of educational opportunities. This leads to a more balanced distribution of educational attainment across genders in the urban marriage market. Additionally, urban areas are more dynamic and diverse, with high population mobility. Urban residents have access to a much larger and more diverse pool of potential partners through various social and professional platforms. In the urban context, the criteria for marriage selection are more multifaceted. While education is still an important factor, personal values, career aspirations, and emotional compatibility also play crucial roles. This means that the influence of education on marriage choices is diluted. Urban women, regardless of their educational levels, have a broader range of options in the marriage market, which results in a more homogeneous pattern of hypogamy across different educational levels.

The observed increase in educational hypogamy reflects significant social changes in China, where traditional gender norms are increasingly challenged by educational and economic developments [57,58]. As women attain higher education and financial independence, they are better positioned to make marital choices that align with personal preferences rather than societal expectations [59]. This shift not only highlights the evolving role of women in modern Chinese society but also underscores the necessity of addressing persistent gender inequalities.

For rural women, the stronger link between education and hypogamy underscores the transformative power of education in regions where traditional norms are deeply entrenched. Education serves as a critical pathway to greater autonomy and broader marital options. However, urban women face a more nuanced interplay of factors, including higher economic demands and greater societal scrutiny, which may complicate their marital decisions despite their educational attainment.

Furthermore, the moderating effects of family background variables such as parental education and social status suggest that intergenerational influences remain potent in shaping marital preferences. This underscores the importance of considering broader social structures when addressing gender and marital dynamics in China.

### Theoretical and practical implications

These findings carry meaningful implications for both theory and practice. Theoretically, this study contributes to the growing literature on assortative mating by demonstrating how women’s educational attainment interacts with contextual variables—such as regional educational disparities, gender norms, income levels, and family background—to shape marriage patterns in a transforming society. It highlights the necessity of incorporating spatial and institutional heterogeneity into models of marital sorting, especially in non-Western contexts like China. The stronger link between education and hypogamy observed among rural women also suggests that educational attainment can serve as a powerful lever for altering entrenched social norms, though its effects are conditioned by structural constraints.

Practically, the revealed patterns of educational hypogamy offer insight into broader societal dynamics. First, they reflect persistent gender asymmetries in education, mobility, and labor market outcomes. Second, they highlight the tension between women’s individual advancement and institutional limitations in both urban and rural marriage markets. These dynamics have implications for gender relations, family structures, and intergenerational mobility, making it crucial for policy frameworks to address the root causes behind these patterns.

To foster more equitable and balanced marital outcomes, a multi – pronged approach is necessary. Policymakers should increase investment in educational infrastructure for rural and disadvantaged male populations to reduce regional mismatches in educational attainment, which expands the partner pool for educated women without reinforcing traditional gender hierarchies. At the same time, expanding employment opportunities, professional training, and mobility programs for educated rural women can enhance their economic autonomy and increase their marital choice set, reducing hypogamous unions driven by constraint.

Moreover, public campaigns and educational curricula need to promote diverse and non – hierarchical notions of marital success to counter the stigma surrounding educational hypogamy, emphasizing compatibility and shared goals over rigid status differences. Additionally, since family background factors like parental education and early – life social class shape women’s marriage outcomes, social policies aimed at reducing intergenerational inequality, such as parenting education, early childhood interventions, and support for low – status families, are crucial. By tackling both structural and normative barriers, these policies can contribute to balanced marriage markets and broader gender equality in Chinese society. Recognizing the complex drivers of educational hypogamy is key to formulating inclusive social policies in a rapidly changing environment.

### Limitations and prospects

While this study provides valuable insights into educational hypogamy in China, it is not without limitations. First, the findings are correlational and do not establish causal relationships between education and hypogamy. Future research could employ longitudinal or experimental designs to better understand these dynamics. Second, the measurement of educational hypogamy relies on years of schooling as a proxy, which may oversimplify the nuanced social value of different educational credentials. For instance, a three-year technical college degree may not be perceived as equivalent to a four-year academic bachelor’s degree in terms of social prestige or labor market value. A more refined classification of education types, or the inclusion of field of study and institutional prestige, would improve the accuracy of hypogamy measurement and interpretation. Third, while the study provides plausible explanations for the stronger association between education and hypogamy among rural women—such as limited local marriage markets and lower female educational attainment—these interpretations remain largely speculative due to the absence of qualitative data. Future studies incorporating qualitative or mixed-methods approaches could yield deeper insight into the cultural and interpersonal dynamics that shape partner selection, particularly in rural settings. Fourth, the analysis focuses on the urban-rural divide but does not capture more granular regional variation that may influence marriage patterns. Local economic conditions, cultural norms, and migration patterns likely play a role in shaping marital decisions and should be explored in future research.

Future research should also explore how these patterns evolve over longer periods and interact with other dimensions of social change, such as economic development, migration, and shifting gender roles. Additionally, examining the potential consequences of educational hypogamy, such as its impact on household dynamics and child-rearing practices, could offer a more comprehensive understanding of its implications for Chinese society.

## Supporting information

S1 File Stata file. (ZIP)
